# Capillary Hemangioma as an Unexpected Pathology in the External Auditory Canal

**DOI:** 10.22038/IJORL.2021.57157.2966

**Published:** 2022-01

**Authors:** Behrouz Barati, Mahboobe Asadi, Fatemeh Jahanshahi

**Affiliations:** 1 *Departmentof Otolaryngology, Taleghani Hospital, Shahid Beheshti University of Medical Sciences, Tehran, Iran.*; 2 *Student Research Committee, Faculty of Medicine, Iran University of Medical Sciences, Tehran, Iran.*

**Keywords:** Capillary, External auditory canal, Hemangioma

## Abstract

**Introduction:**

Hemangiomas are benign vascular lesions frequently observed in infancy and childhood.

**Case Report:**

A 14-year-old boy was referred to the hospital with a left-sided ear canal mass and hearing impairment. Otoscopic examination revealed a mass that occluded the canal. The transcanal surgical excision was performed. The mass was dissected from the skin, no bony erosion was noted intraoperatively, and the skin was returned back properly. The histopathological report demonstrated a capillary hemangioma of the external auditory canal.

**Conclusions:**

Hemangiomas are relatively common in the head and neck, but rarely detected in the external ear canal and tympanic membrane. Hemangioma of the ear canal may be asymptomatic and accidentally observed. Despite the rarity of the mass, it is important to consider them in the differential diagnosis of external auditory canal masses. The computed tomography and magnetic resonance imaging with gadolinium help to reveal the vascular nature of the mass. Complete surgical excision is the treatment of choice.

## Introduction

Hemangiomas are recognized as benign vascular lesions in infancy and childhood mostly observed in head and neck areas. One-third of these tumors may present at birth. Hemangiomas of the external auditory canal and eardrum are rare conditions. It initially presents as a small vascular lesion in the deep posterior bony external auditory canal or at the posterosuperior tympanic membrane. It is usually asymptomatic till becomes bigger or bleeds due to the manipulation by the patient. Hemangiomas are classified into different histological categories: cavernous, capillary, and mixed. So far, only a few cases of capillary hemangiomas of the external auditory canal have been reported (1,2).

## Case Report

A 14-year old adolescent boy was referred to our otolaryngology clinic with a complaint of right-sided ear canal painless mass and hearing impairment. Based on the patient's statement, he experienced progressive hearing loss for one month to the extent that he had difficulty hearing. He also stated that the mass which was small at first grew fast and filled the ear canal. He denied any history of ear discharge, pain, tinnitus, and vertigo. According to the patient, the earlier attempts for mass excision were followed by the recurrence of the problem after a few weeks. He did not mention any other disease or drugs administration in his medical history. Moreover, no benign or malignant cysts or tumors were reported in his family members.

**Fig 1:**
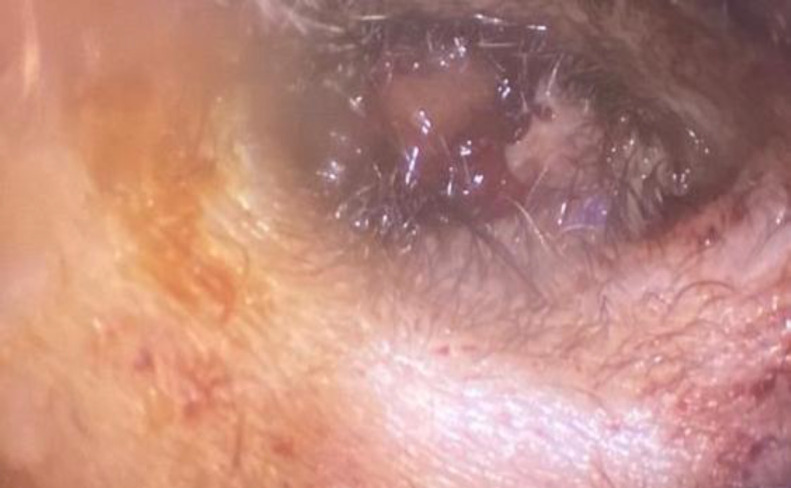
Mass arose from posterosuperior wall of external auditory canal under the skin and clogged the canal

On otoendoscopic examination, a broad base mass was detected which arose from the posterosuperior wall of the external auditory canal under the skin and clogged the canal (Figure 1).

**Fig 2:**
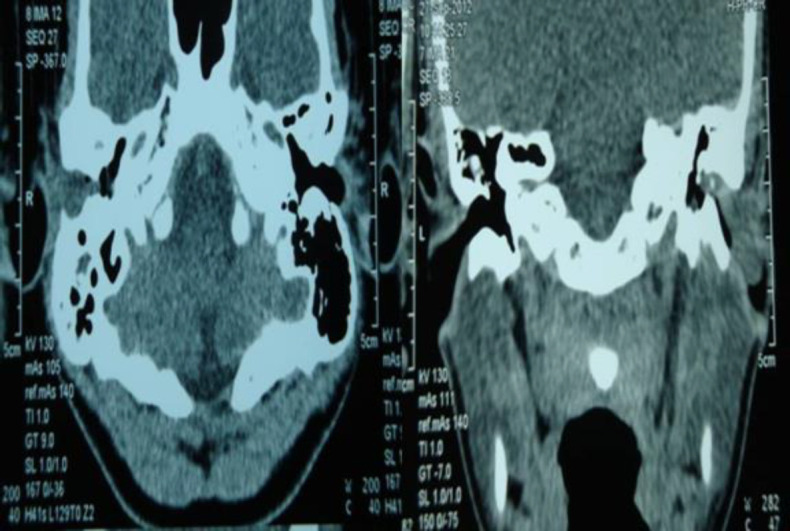
CT scan in axial and coronal view, showing soft tissue mass in the external auditory canal without bony erosion or involvement of the tympanic membrane

**Fig 3:**
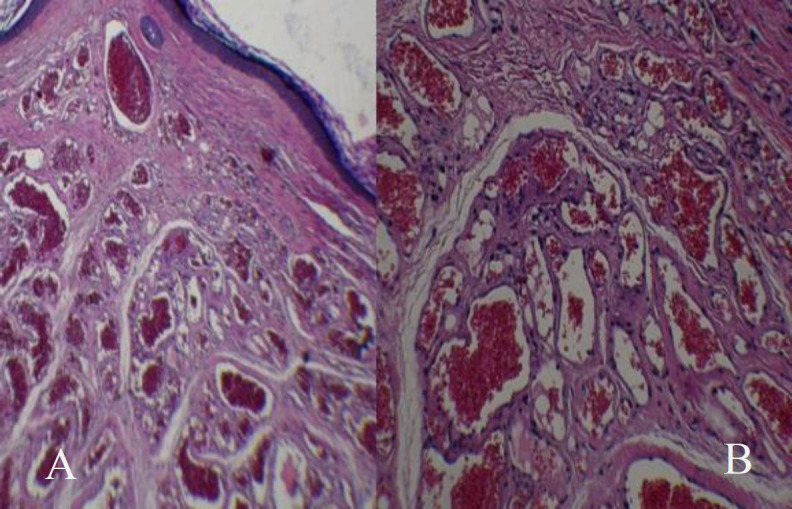
A, Histopathology of tumor at low-power magnification (x10; haematoxylin and eosin stain), displaying well-defined vascular tumor composed of arranged small capillaries which are separated by scant connective tissue stroma

The mass was consistently soft, without any ulcer, discoloring, or bleeding on touching. The probing demonstrated no connection between mass and tympanic membrane; moreover, the eardrums were intact. The vascular appearance of the lesion was suggestive of the provisional diagnosis of granulation tissue and vascular tumor. Therefore, after examination, the radiological investigation was requested and Computed Tomography (CT) scan demonstrated a smooth mass outside the tympanic membrane (Figure 2,3). 

B, High-power magnification (x40; haematoxylin and eosin stain) of vascular tumor, depicting closely packed blood-filled capillaries lined by monolayer flat endothelial cells.

No evidence of bony erosion was noted, the middle ear cavity and the ossicles looked normal, and no middle ear lesions were observed. The pre-operative audiogram illustrated unilateral moderate conductive hearing loss.The patient with an initial diagnosis of vascular tumor underwent general anesthesia and the transcanal surgical excision at the specialist discretion. The mass was dissected from the skin and sent to the pathologist, no bony erosion was noted intraoperatively, and the skin was returned back properly with no defect. The tympanic membrane was kept intact. According to the pathological investigation results, the mass was a capillary hemangioma in the external auditory canal.Finally, the patient was discharged in good general health after a successful recovery without any side effects. Postoperatively, the site of surgery healed well and the conductive hearing loss was disappeared. The postoperative audiogram recorded normal hearing ability one month after the surgery. Furthermore, no significant mass was observed on post-operative otoendoscopic examination. In addition, a one-year clinical follow-up demonstrated no recurrence of the lesion and symptoms.

## Discussion

The external auditory canal is a segment of the external ear consisting of two parts of inner and outer. The inner ear is bony and surrounded by temporal, tympanic, and mastoid bone, while the outer ear is cartilaginous, leads to the auricle, and has thick skin with hair follicles, sebaceous glands, and ceruminous glands. The lesions of the external auditory canal can be classified as benign and malignant lesions according to their severity. Benign lesions have a higher prevalence than malignant ones (3).Hemangioma is a benign vascular tumor which appears in the skin, soft tissue, and bone. It has two growth phases: the first phase is rapid proliferation, while the second one is a regressive phase in which mass gradually shrinks and disappears (4). 

Therefore, hemangioma usually forms at birth, mostly passes the regressive phase, and disappears within the first year of life. Nonetheless, those cases which do not complete the second phase may become symptomatic and should undergo invasive investigation (1). 

Histologically, there are different types of hemangiomas, including cavernous, capillary, and mixed types. Hemangiomas are relatively common in the head and neck; nonetheless, they are very occasionally observed at the external ear canal and tympanic membrane (5). The review of the literature revealed that this is the second case of ear canal hemangioma reporting in childhood. The hemangiomas may arise from either the lamina propria of the eardrum (6) or the skin of the external auditory canal, specifically from the posterosuperior wall. No evidence of bony erosion has been referred to in the literature (7).

The etiology of hemangioma in the ear canal is not well recognized; however, previously conducted studies claimed that some predisposing factors, such as trauma, infection, gender, and hormonal factors, can affect its formation and development (4,8). The histopathological characteristic of hemangioma is the proliferation of vessels with dilation which is arranged close to each other. These vessels have a single layer of flat endothelium (5). Hemangiomas of the ear canal may be asymptomatic and accidentally diagnosed. Moreover, they can be associated with ear fullness, conductive hearing loss, ear bleeding, pulsatile tinnitus, otorrhea, pediculated auricular mass, and red-purple tympanic membrane (9). 

Apart from radiologic investigations, the temporal bone CT scan is the first choice to assess these types of tumors, providing useful information on the presence of any bony erosion. 

Hemangioma has the same density as brain parenchyma in CT scan results; moreover, magnetic resonance imaging (MRI) can be performed to precisely differentiate the vascular lesions. Hemangiomas present as intermediate signal T1 and hyper signal T2 in MRI. The angiography with embolization is recommended for advanced lesions (10).

Differential diagnosis of hemangioma of the external auditory canal includes squamous cell carcinoma, basal cell carcinoma, spindle cell carcinoma, amelanotic melanoma, true hemangioma, and common warts. 

The treatment is complete surgical excision through a trans-canal, end-aural, or posterior auricular approach. Nevertheless, embolization or radiation therapy may be recommended in special situations (11). Recurrence probability in these cases after surgery is about 20%; however, incomplete excision or the re-injury of the previous lesion area can double this probability (8).

## Conclusion

Although external auditory canal hemangiomas are rare, it is important to consider them in the differential diagnosis of external auditory canal masses.  Contrast-enhanced CT or MRI with gadolinium helps to reveal the vascular nature of the mass. The biopsy of vascular tumors, such as hemangioma, is not recommended since it may cause excessive bleeding. Complete surgical excision is the best choice of treatment with a minor risk of hearing impairment.
